# Medication Adherence and Treatment-Resistant Hypertension in Newly Treated Hypertensive Patients in the United Arab Emirates

**DOI:** 10.3390/jcm10215036

**Published:** 2021-10-28

**Authors:** Akshaya Srikanth Bhagavathula, Syed Mahboob Shah, Elhadi Husein Aburawi

**Affiliations:** 1 Institute of Public Health, College of Medicine and Health Sciences, UAE University, Al Ain 17666, Abu Dhabi, United Arab Emirates; 201890132@uaeu.ac.ae (A.S.B.); syeds@uaeu.ac.ae (S.M.S.); 2 Department of Pediatrics, College of Medicine and Health Sciences, UAE University, Al Ain 17666, Abu Dhabi, United Arab Emirates

**Keywords:** blood pressure, hypertension, medication adherence, treatment-resistant hypertension, control, United Arab Emirates

## Abstract

(1) Background: The present study aimed to analyze medication adherence and its effect on blood pressure (BP) control and assess the prevalence of treatment-resistant hypertension (TRH) among newly treated hypertensive patients in the United Arab Emirates (UAE); (2) Methods: A retrospective chart review was conducted to evaluate 5308 naïve hypertensive adults registered for the treatment across Abu Dhabi Health Services (SEHA) clinics in Abu Dhabi in 2017. After collecting data regarding basic details and BP measurements, patients were followed up for six months. Patients who did not reach BP targets despite taking three or more antihypertensive medications were defined as TRH; (3) Results: The overall adherence to antihypertensive treatment was 42%. At 6-month, a significant reduction in BP was observed in patients adherent to medications (systolic: −4.5 mm Hg and diastolic: −5.9 mm Hg) than those who were nonadherent to antihypertensive therapy (1.15 mm Hg and 3.59 mm Hg). Among 189 patients using three or more antihypertensive medications for six months, only 34% (*n* = 64) were adherent to the treatment, and only 13.7% (*n* = 26) reached the BP target. The prevalence of TRH was 20.1%; (4) Conclusions: Medication adherence and BP control among the participants were suboptimal. The study shows a high prevalence of TRH among newly treated hypertensives in the UAE. More extraordinary efforts toward improving adherence to antihypertensive therapy and more focus toward BP control and TRH are urgently needed.

## 1. Introduction

Uncontrolled hypertension is considered one of the most important risk factors for cardiovascular disease (CVD) [1]. Numerous efficacious medications are currently available for treating hypertension. However, nonadherence to antihypertensive treatment is among the major causative factors for uncontrolled hypertension [2,3,4,5]. The consequences of lack of adherence to an antihypertensive drug regimen can significantly increase risks for cardiac and cerebrovascular diseases [6,7,8,9,10], poorer health outcomes [11], and increased healthcare costs [12]. Hypertension guidelines have strongly emphasized the importance of medication adherence to improve blood pressure (BP) control [13,14].

Treatment-resistant hypertension (TRH) is defined as uncontrolled hypertension (office BP >130/80 mm Hg or >140/80 mm Hg) despite using three different classes of antihypertensive medications, including a diuretic, at their maximally tolerated doses [15,16,17]. In general, patients adherent to ≥3 classes of antihypertensive medications but not reaching the BP goals are called TRH. A similar scenario in nonadherent patients is considered pseudo-resistant hypertension (PRH) [18]. Poor adherence to antihypertensive drug therapy is an important cause of apparent TRH and has a more significant impact on patients’ well-being. However, most studies were incapable of ruling out PRH caused by nonadherence to drug therapy and distinguishing true TRH from PRH [19]. Therefore, knowledge regarding medication adherence after initiation of therapy, TRH among the patients adherent to antihypertensive medications, and PRH caused by poor adherence are essential to improve hypertension management.

In the Middle East, prior studies on hypertension had certain limitations, including lack of longitudinal BP data, failure to apply a uniform definition of hypertension, questionnaires to assess medication adherence, and no studies focused on TRH. Furthermore, epidemiological data on adherence to antihypertensive medications, its influence on BP control, and the prevalence of TRH in newly treated hypertensive patients are sparse.

Accordingly, we assessed adherence to the medication and its effect on BP control among patients with newly treated hypertension based on prescriptions filled for hypertension medication, BP measurements, and adherence data in the United Arab Emirates (UAE). Then, the prevalence of TRH and PRH was assessed among a subset of patients treated with three or more antihypertensive medications for six months and not reaching BP goals.

## 2. Materials and Methods

### 2.1. Study Cohort

**Figure 1:**
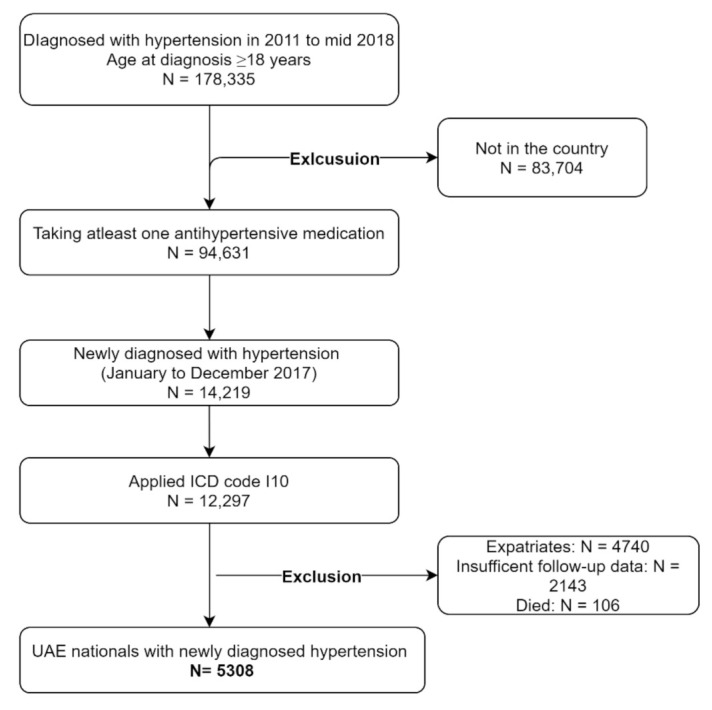
Consort flow diagram of the cohort studies.

Hypertensive patients comprising 178,335 aged 18–100 years registered for the treatment across SEHA facilities from 2011 to June 2018 were identified. Of these, those who are taking at least one antihypertensive medication from 1 January 2011, until 31 December 2016, were identified. Those newly diagnosed patients and first dispensed of an antihypertensive medication from 1 January 2017, were considered index date. To ensure that selection only included newly-treated individuals, patients for whom one or more prescriptions of hypertensive medications or hospitalization due to hypertension before the index date were excluded. A total of 12,297 patients with incident hypertensive patients and on treatment were identified through ICD-10 code I10 (essential hypertension) as primary diagnoses were considered. Finally, because the purpose of the study was to determine the medication adherence and its effect on BP control in the UAE nationals, patients with insufficient follow-up data (*n* = 2143), expatriates (*n* = 4740), and died (*n* = 106) were excluded. The remaining patients constituted the study cohort. More details are shown in Figure 1.

### 2.2. Study Setting

The data for the study were collected from the chronic disease clinics and included cardiology, endocrinology, and nephrology clinics across SEHA facilities in the UAE. SEHA is the largest and main corporate healthcare network in the UAE and provides comprehensive medical care to five million population. Throughout the Abu Dhabi region, 38 SEHA health clinics are located across primary and secondary care centers and an additional 14 auxiliary clinics are available at tertiary care centers [20]. 

### 2.3. Study Population

The study sample was identified through a retrospective chart review of newly treated hypertensive adults registered for antihypertensive treatment between 1 January 2017, and 31 December 2017. Each patient was retrospectively followed up for six months to evaluate their outcomes and other characteristics. The chart review was conducted from 1 September 2018, until 31 October 2019. The study included patients diagnosed by a consulting physician using the 24 h out-of-office ambulatory BP monitoring (ABPM) or home-based BP (HMBP) measurements and started subsequent antihypertensive treatment. All the Abu Dhabi health services (SEHA) facilities are equipped with automated office BP devices, in which the patient is resting alone in the examination room, and BP is measured using an automated device that gives the average of several measurements. According to the SEHA protocol, the physician should use the automated office BP device to initiate the therapy or refill the medications. For the current study, we used the data of automated office BP obtained at baseline and 6-month follow-up. Patients with a history of using antihypertensive medication or hospitalization due to elevated BP in the past 12 months of the index period were excluded.

### 2.4. Data Source

We conducted the study using the Cerner^®^ health care databases managed by SEHA facilities. These extensive, electronically linked health data pertained to the UAE residents covered by provincial health insurance [21]. Each patient’s electronic medical records (EMR) were systematically reviewed and the collected information was carefully cross-checked with the patients’ Financial Identification Number (FIN) for pharmacy claims linked to the medical record number (MRN) for each patient.

### 2.5. Inclusion and Exclusion Criteria

The inclusion criteria were:

- UAE national representative patients.
- Aged ≥ 18 years.
- Newly diagnosed with hypertension during 2017.
- Registered for hypertension treatment at SEHA facilities.

Exclusion criteria

- Non-UAE nationals.
- Patients with established hypertension and not confirmed by ABPM or HBPM by treating physician.
- Hospitalized hypertension patients.
- Not on antihypertensive medications.
- Not on follow-up for at least one-month treatment.
- Hypertensive patients with a history of antihypertensive medication use or hospitalization due to elevated BP in the past 12 months of the index period.
- Patients with hemoglobinopathy disorders and/or with systemic or malignant disease (needs special consideration).
- Significant renal impairment (plasma creatinine concentration >2.0 mg/L).

### 2.6. Operational Definitions

According to international guidelines on detection, evaluation, and treatment of high blood pressure, hypertension was defined as a threshold for systolic/diastolic BP of ≥130/80 mm Hg [14] or ≥140/80 mm Hg for ≥65 years old [15,16,17].

Medication adherence was determined for each patient based on adherence (proportion of days covered based on pharmacy refill information) to each antihypertensive medication during the study period and the average of these medications. Patients with a summary adherence measure of ≥80% were considered medication adherent [22,23,24]. 

TRH is defined as uncontrolled BP despite using three different classes of antihypertensive medications, including a diuretic, at their maximally tolerated doses [15,16,17]. The study cohort patients took three or more medications for at least one month and were followed up for six months to assess adherence to antihypertensive medications. Patients adherent to treatment but not reaching the BP goal were considered TRH [13,15,16,25]. In contrast, resistant hypertension caused by poor medication adherence was considered PRH [18,21,26]. 

### 2.7. Outcome Measures

The study’s primary outcomes include adherence to antihypertensive treatment, changes in the BP from baseline to six months, and BP control at 6-month follow-up. In the secondary analysis, we determined the prevalence of TRH in patients adherent to medications and PRH in patients nonadherent to antihypertensive treatment. 

### 2.8. Data Collection

Data collected at baseline line included age, sex, healthcare center location (rural/urban), type of health facility (primary, secondary, and tertiary), smoking status (smoker/non-smoker), body mass index (BMI, kg/m^2^), and diabetes mellitus (DM) status. Along with the BP parameters, the data collected at baseline and sixth month included the number and type of antihypertensive drugs prescribed and pharmacy refilling information. 

### 2.9. Statistical Analysis

All statistical analyses were performed using IBM SPSS statistics software for Windows, version 24.0 (IBM Corp, Armonk, NY, USA). The baseline characteristics were presented as frequency and percentages for categorical variables. The mean ± standard deviation (SD) was used for continuous variables. The chi-square test was used to compare the differences between categorical variables, and ANOVA or the Kruskal–Wallis test was used for continuous variables, as appropriate. A Wilcoxon signed rank-sum test was used to determine if a significant difference between baseline and at six months across various categories. Factors independently associated with the changes in BP at six-month follow-up between adherent and nonadherent groups were determined using the Mann–Whitney U test or Kruskal–Wallis test, as appropriate. Multivariable logistic regression analyses were performed to identify the factors associated with adherence to antihypertensive medications. Crude and adjusted odds ratios (OR) with 95% confidence intervals (CI) were calculated. Adjustments were performed for multiple covariates (age, type of health center, BMI, smoking). A two-sided *p*-value of <0.05 was considered statistically significant. A decision tree algorithm was used to determine the prevalence of TRH and PRH in the study cohort.

## 3. Results

A total of 5308 patients with incident hypertension started on antihypertensive therapy. Among these patients, the majority (77.6%) had taken one antihypertensive medication, whereas 189 (3.6%) patients had taken three or more classes of antihypertensive medications concurrently for at least one month. At baseline, subjects had a mean (±SD) SBP of 133.9 ± 17.1 mm Hg and DBP of 72.9 ± 12.7 mm Hg. Higher medication use was significantly associated with old age. The baseline characteristics of the study cohort are listed in Table 1.

At 6-month follow-up, a significantly higher BP reduction was observed in men (SBP: −1.68 mmHg and DBP: −1.15 mmHg) than women (SBP: −0.84 mmHg), and no significant reduction in the DBP (0.22 mmHg) was observed in women. Moreover, a significant reduction in the SBP was observed in the 41–75 years age group, and those aged 61–75 years showed higher SBP difference from baseline (−1.82 mmHg) than other age groups. Those hypertensives treated at primary care settings showed a significant reduction in the SBP by −2.31 mmHg (*p* < 0.001) and DBP by −2.15 mmHg (*p* < 0.001), with no such reduction in SBP and DBP in those treated at secondary and tertiary care settings, as shown in Table 2.

After six months of treatment initiation, adherence to antihypertensive medication was found to be 42%. A significantly higher adherence was found among the patients taking one medication (42.8%) than those taking three or more medications (34%). The level of adherence to the number of antihypertensive drugs used was stratified by gender, age, type of healthcare setting, smoking status, BMI, and DM status, as presented in Table 3. Overall, medication adherence was higher among patients younger than 40 years (67.9%), DM (53.6%), treated at secondary care settings (49.1%), non-smokers (47.8%), and with a normal BMI of <25 kg/m^2^ (47.1%). However, significant differences in medication adherence were observed by gender, health center location, type of settings, and DM status.

**Figure 2:**
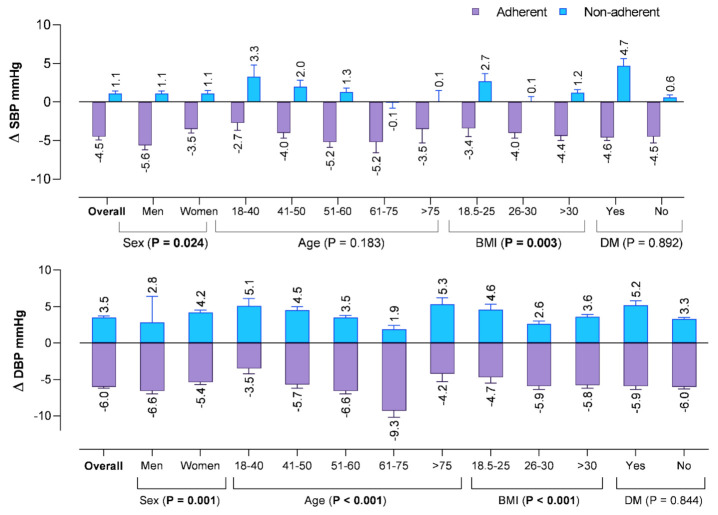
Mean changes in the blood pressure from baseline at 6-months in the subjects according to their adherent status. Significant factors were bolded.

In analyzing BP changes at six-month follow-up, a significant mean reduction in the SBP of 4.5 mm Hg (95% CI: −5.4–−3.77). and DBP of 6.0 mm Hg (95% CI: −6.5–−5.4) was observed among those who were adherent to antihypertensive treatment compared to the nonadherent patients (SBP: 1.15 mm Hg, 95% CI: 0.50–1.79, DBP: 3.57 mm Hg, 95% CI: 3.14–4.04) (*p* < 0.001 for SBP and *p* < 0.001 for DBP). Likewise, when comparing BP changes among patients adherent to medications at 6-month follow-up, a significant higher SBP reduction was observed in men than women (– 5.6 mm Hg versus–3.5 mm Hg; *p* = 0.024), and in patients with BMI of >30 kg/m^2^ (−4.4 mm Hg; *p* = 0.003), as shown in Figure 2. Moreover, significant (*p* ≤ 0.001) differences in DBP were observed across age, sex, and BMI; however, no such differences in SBP and DBP were observed in patients with or without DM.

**Figure 3:**
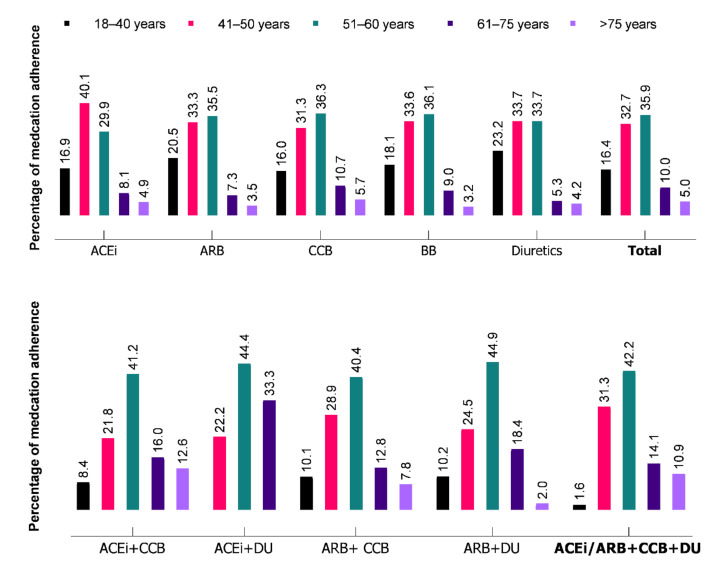
Medication adherence to different classes of drugs during the first 6-months of antihypertensive treatment initiation. ACEi: angiotensin-convertase enzyme inhibitor, ARB: angiotensin-receptor blockers, CCB: calcium channel blockers, BB: beta-blockers, DU: diuretics.

As shown in Figure 3, optimal adherence was observed in middle-aged categories (51–60 years) for all the different classes of antihypertensive drugs. Moreover, younger age (18–40 years) and older age (>60 years) had low adherence to antihypertensive therapies irrespective of the number of medications prescribed. 

Among these newly diagnosed diseases, the overall medication adherence was higher in 51–60 years age group, particularly combination therapy of Angiotensin-receptor blockers (ARBs) or angiotensin-converting enzyme (ACEi) with diuretics (DU) (45% and 44.4%), and ACEi with calcium channel blockers (CCBs) (44.4%). In contrast, adherence to ACEi in the 40–50 year age group was higher (40.1%) and it gradually decreased as the age increased with the adherence being at 29.9% and 13% in the 51–60 years and >60 years age group, respectively. Medication adherence to three or more antihypertensive medications was higher in the 51–60 year age group (42.2%) than 41–50 years (31.3%), 61–75 years (14.1%), and > 75 years (10.9%). 

Factors significantly associated with the number of medications and adherence to antihypertensive drugs are shown in Table 4. The unadjusted logistic regression analysis indicated that treatment at secondary care settings (OR: 1.35, 95% CI: 1.10–1.65), non-smokers (OR: 2.07, 95% CI: 1.83–2.37), and patients with DM (OR: 1.73, 95% CI: 1.47–2.03) were significant predictors of adherence when a single antihypertensive drug was taken. Similar findings were observed when two medications were taken (except in secondary care setting). On the contrary, non-smokers (OR: 2.30, 95% CI: 1.22–4.35) and patients with DM (OR: 4.57, 95% CI: 1.63–12.85) were associated with adherence to three or more antihypertensive drugs.

After adjusting for multiple covariates, an increase in age and BMI was associated with a gradual decrease in odds to adherence to one and two antihypertensive medications. Among the patients receiving monotherapy, men (OR: 1.20, 95% CI: 1.04–1.39), patients treated at secondary care setting (OR: 1.45, 95% CI: 1.15–1.84), and DM patients (OR: 1.27, 95% CI: 1.06–1.53) were more likely to be adherent to monotherapy. Furthermore, DM patients were 3.6 times more likely to adhere to three or more antihypertensive medications (OR: 3.61, 95% CI: 1.11–11.72).

**Figure 4:**
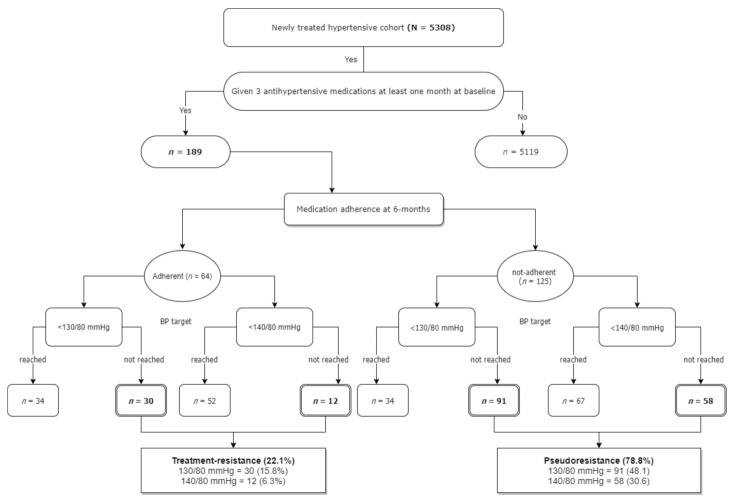
Flow chart of study population taking three or more medications for at least 1 month and prevalence of treatment-resistant hypertension.

Figure 4 presents the 5308 patients in detail based on their baseline BP, follow-up at 6-month, and subsequent TRH and PRH status. Among a total of 189 patients taking three or more classes of antihypertensive medications for at least one month, 64 (33.8%) patients were adherent, and 125 (66.2%) were nonadherent to therapy. Among the patients adherent to medications, 28 (14.8%, 95% CI: 10.0–20.7) with the age of <65 years did not reach the BP target of 130/80 (mean BP: 119.5 ± 7.61 mmHg), and ten (5.3%, 95% CI: 2.5–9.5) with the age of ≥65 years had BP above 140/80 (151.03 ± 13.1 mmHg). The overall prevalence of TRH was 20.1% (95% CI: 14.6–26.5). On the contrary, 125 patients were nonadherent to antihypertensive treatment, 21 (11.1%, 95% CI: 7–16) and 25 (13.2%, 95% CI: 8.7–18.9) patients did not reach the BP target of 130/80 (mean BP: 146.7 ± 8.1 mmHg), and 140/80 (mean BP: 151.0 ± 8.2 mmHg, respectively. These patients were classified as PRH (24.4%, 95% CI: 18.4–31.1) caused by poor medication adherence.

The mean SBP in patients with TRH was higher in those aged ≥ 65 years (151.0 ± 8.2 mmHg) than those aged <65 years (146.5 ± 13.8 mmHg). For PRH patients, the mean SBP and DBP were much higher in the older population aged ≥65 years than in younger patients (<65 years). More details are in Table 5. 

## 4. Discussion

In clinical practice, nonadherence is a common cause of antihypertensive treatment failure and treatment resistance. In the current study, of the 5308 patients with incident hypertension who started on hypertension treatment, adherence to all antihypertensive medications was found among 42% of the patients. Meanwhile, 1.6% of patients developed resistant hypertension within 6-months of follow-up. Poor medication adherence (34%) was detected in the patients taking three or more antihypertensive agents. Patients treated at secondary care settings and male gender were more likely to be adherent to monotherapy. A diagnosis of DM was found to be associated with higher odds of treatment adherence to monotherapy and three or more antihypertensive drugs. However, an increase in age and BMI was associated with lower odds of medication adherence.

One of the most important contributions of this study is to estimate the level of medication adherence and the prevalence of TRH. To the best of our knowledge, this study is the first to determine that among newly treated hypertensive patients, one out of two patients would be nonadherent to antihypertensive medications, and one out of five taking three or more medications may have TRH. Studies have reported that adherence to pharmacotherapy for hypertension after treatment initiation is typically at <50% [2,8,10,22,27]. A recent meta-analysis reported that the overall nonadherence to antihypertensive medications in the Asian population was 48% (95% CI: 41–54%) and 41% for the Middle Eastern population (95% CI: 30–52) [27]. The rate of TRH in our study was much higher than a study conducted in Israel that reported a lower prevalence of TRH (2.2%) among patients with uncontrolled hypertension [28]. 

Current estimates on the prevalence of adherence to antihypertensive medication in the Middle East are based on cross-sectional studies and used self-reported questionnaires [27]. The reported proportion of medication adherence to antihypertensive therapy in this study (42%) is in concordance with adherence rates reported in Arabian Gulf countries, including Saudi Arabia (40%) [29], Oman (49%) [30], and two studies in the UAE (52% and 54%) [31,32]. This study has the advantage of accounting for longitudinal BP control and medication adherence using detailed pharmacy information among a large, community cohort of patients with incident hypertension.

Another important finding of this study is the evaluation of the BP changes in patients adherent to antihypertensive treatment compared to the nonadherent patients. Focusing on BP changes is important to understand the implications of medication adherence on BP control in incident hypertensive patients. Analysis of BP changes indicated that patients adherent to treatment had a significant reduction in BP than those nonadherent to antihypertensive therapy (Figure 2). The decline in SBP and DBP was significantly higher in men (5.6 mm Hg and 6.6 mm Hg) and those aged 61–75 years (5.2 mm Hg and 9.3 mm Hg). This numeric difference in BP in our study cohort indicates a clinically meaningful effect of adherence on BP control. Thus, monitoring medication adherence on each visit can be used as a therapeutic tool and significantly improve BP. Educating patients regarding positive changes in diet and lifestyle in standard care coupled with improved adherence to antihypertensive medication can have an absolute improvement in BP control. 

Patients with TRH have a poor prognosis and a higher risk for end-organ damage, heart failure, cardiovascular events, and premature death [15,16,17,18,19]. Few studies described the prevalence of TRH in incident hypertensive patients [19,23,24,33], and a meta-analysis by *Noubiap* et al. reported the pooled prevalence of 14.7% (95% CI: 13.1–16.3) and PRH of 10.3% (95% CI: 6.0–15.5) [33]. In our study, the overall prevalence of TRH and PRH was 20.1% and 24.4%, respectively. Based on the present study, one in five incident hypertensive patients taking three or more hypertensive medications will continue to meet TRH criteria over follow-up. In addition, we have shown that one in four patients who were not adherent to hypertensive treatment had PRH. According to the guidelines-recommended BP targets for different age groups, the prevalence of TRH among patients aged 18–64 years was 14.8% and 5.3% in patients aged 65 years or older. Our overall estimates of TRH (20.1%) are higher than global prevalence (14.7%) [33], but lower than Europe (32%) [34], Pakistan (32%) [35], and Poland 25% [36]. The discrepancy is significant, and the variation might be largely due to the differences in study design, target population, definitions used, and failure to exclude suboptimal adherence, a common cause of apparent TRH. A recent randomized trial on smartphone app intervention showed improved patient adherence and BP control in patients with uncontrolled hypertension [37].

Moreover, the findings of this study demonstrated that an increase in age and BMI was associated with relatively poor medication adherence. Many studies advocated these findings and indicated that baseline BP, age, gender, ethnicity, income, and other socioeconomic indicators had been linked to nonadherence and TRH [38,39,40,41]. Therefore, early identification of causes of nonadherence to antihypertensive treatment, testing motivational interviews to increase patient engagement, and their impact on patients’ medication-taking behavior should be investigated in the future.

This study has some limitations. First, the present study relied on automated office BP measurements and pharmacy refill information from an electronic medical record; however, this method for determining hypertension and medication adherence has been widely applied in previous studies [18,21,23,41]. Second, previous studies have indicated that ABPM may provide more accurate estimates of TRH and be more prognostic than routinely used office-based BP measurement [42,43,44,45]. However, office-based BP measurements are routinely used in the management of hypertension. Third, we did not account for optimal dosage of medications or the use of fixed-dose combination therapy; however, medication use and dosage in the present study represent real-world management choices. Fourth, due to the study’s retrospective nature, we could not distinguish the white-coat effects on apparent TRH, which might present a chance of misclassification as some patients may use additional medications and be treated at private health facilities. Fifth, although the adherence was poor, we did not investigate the potential causes and barriers to antihypertensive medication adherence. Also, we could not collect data on several variables, such as diet, salt intake, physical activity, and restriction of calories that can impact the BP control. Last, the study was conducted across 54 SEHA health facilities in the emirates of Abu Dhabi; still, it is difficult to generalize the results to the whole UAE population.

## 5. Conclusions

In the cohort of 5308 patients with incident hypertension, both medication adherence and BP control were suboptimal. More than half of the patients were nonadherent to treatment. The patients who were adherent to an antihypertensive therapy reported a significant BP reduction at a 6-month follow-up. Age and BMI were among the independent risk factors for medication adherence. One in five patients taking three or more antihypertensive medications continued to meet the criteria for TRH. These findings support the need for more extraordinary efforts toward improving medication adherence and BP control during the early stages of hypertension. Further studies are needed to assess the causes of nonadherence to antihypertensive treatment and the prognosis of patients with TRH. 

## Figures and Tables

**Table 1:** Clinical characteristics of study cohort with number of antihypertensive medications given at baseline.

Characteristics	Total (N = 5308)	Number of Antihypertensive Medications			*p*-Value *
		One (4117, 77.6%)	Two (1002, 18.9%)	Three (189, 3.6%)	
Age, Mean ±SD	54.8 ± 11.5	54.0 ± 11.5	57.1 ± 11.2	59.3 ± 11.4	<0.001
Gender, *n* (%)					0.201
Men	2459 (46.3)	1906 (35.9)	454 (8.6)	99 (1.9)	
Women	2849 (53.7)	2211 (41.7)	548 (10.3)	90 (1.7)	
Health center location, *n* (%)					0.970
Rural	2595 (48.9)	2012 (37.9)	492 (9.3)	91 (1.7)	
Urban	2713 (51.1)	2105 (39.7)	510 (9.6)	98 (1.8)	
Healthcare setting, *n* (%)					0.126
Primary	3189 (60.1)	2494 (47)	582 (11)	113 (2.1)	
Secondary	795 (15.0)	588 (11.1)	176 (3.3)	31 (0.6)	
Tertiary	1324 (24.9)	1035 (19.5)	244 (4.6)	45 (0.8)	
Smoking, *n* (%)					0.001
Smoker	1883 (35.5)	1406 (26.5)	393 (7.4)	84 (1.6)	
Non-smoker	3425 (64.5)	2711 (51.1)	609 (11.5)	105 (2.0)	
BMI (kg/m^2^), (*n* = 4583, 86.3%)	31.2 ± 7.4	31.2 ± 7.6	31.0 ± 6.5	31.2 ± 6.9	0.824
<18.5	41 (0.9)	32 (0.7)	8 (0.2)	1 (0)	
18.5–25	631 (11.9)	500 (10.9)	114 (2.5)	17 (0.4)	
25–30	1485 (28)	1148 (25)	280 (6.1)	57 (1.2)	
>30	2483 (45.7)	1889 (41.2)	454 (9.9)	83 (1.8)	
Diabetes, *n* (%)					0.001
No	4,437 (83.6)	3403 (64.1)	863 (16.3)	171 (3.2)	
Yes	871 (16.4)	714 (13.5)	139 (2.6)	18 (0.3)	
Baseline systolic BP, mmHg	133.9 ± 17.1	134.0 ± 17.1	133.8 ± 17.2	134.4 ± 18.4	0.962
Baseline diastolic BP, mmHg	72.9 ± 12.7	73.1 ± 12.6	72.2 ± 12.9	71.9 ± 12.9	0.061

* chi-square test for categorical variables and ANOVA or Kruskal–Wallis test for continuous variables.

**Table 2:** Blood pressure at baseline and at 6-month according to the subgroups.

Characteristics	Systolic Blood Pressure				Diastolic Blood Pressure			
	Baseline	6-Months	Absolute Difference	*p*-Value *	Baseline	6-Months	Absolute Difference	*p*-Value *
Overall	133.9 (133.5–134.4)	132.7 (132.3–133.2)	−1.23 (−1.70 to −0.67)	<0.001	72.9 (72.6–73.3)	72.5 (72.1–72.8)	−0.41 (−0.80 to −0.27)	<0.001
Sex								
Men	133.8 (133.1–134.5)	132.1 (131.5–132.8)	−1.68 (−2.46 to −0.91)	<0.001	74.3 (73.8–74.8)	73.2 (72.7–73.6)	−1.15 (−1.69 to −0.60)	<0.001
Women	134.1 (133.4–134.7)	133.2 (132.6–133.8)	−0.84 (−1.57 to −0.12)	0.046	71.7 (71.2–72.1)	71.9 (71.4–72.3)	0.22 (−0.29 to 0.73)	0.795
Age (years)								
18–40	134.7 (133.2–136.1)	133.3 (132.5–134.1)	−0.81 (−2.48 to 0.85)	0.262	75.2 (74.0–76.4)	74.5 (73.4–75.7)	−0.72 (−1.96 to 0.52)	0.289
41–50	133.2 (132.3–134.1)	131.8 (130.9–132.7)	−1.39 (−2.46 to −0.32)	0.013	75.3 (74.6–75.9)	74.0 (73.3–76.7)	−1.25 (−2.03 to −0.47)	0.002
51–60	134.0 (133.3–134.7)	133.0 (132.3–133.7)	−1.03 (−1.83 to −0.22)	0.006	72.6 (72.1–73.1)	72.5 (72.0–73.0)	−0.09 (−0.64 to 0.45)	0.244
61–75	134.5 (133.4–135.7)	132.7 (131.6–133.8)	−1.82 (−3.15 to −0.49)	0.016	71.6 (70.8–72.4)	70.8 (70.1–71.5)	−0.81 (−1.74 to 0.11)	0.104
>75	133.5 (131.6–135.5)	132.5 (130.6–134.4)	−1.07 (−3.30 to 1.15)	0.246	65.8 (64.5–67.1)	67.9 (66.6–69.3)	2.17 (0.63 to 3.70)	0.027
Health center location								
Rural	133.9 (133.2–134.5)	132.8 (132.1–133.4)	−1.11 (−1.87 to −0.35)	0.004	73.0 (72.5–73.5)	72.5 (72.1–73.0)	−0.46 (−0.99 to 0.06)	0.039
Urban	134.0 (133.4–134.7)	132.7 (132.0–133.3)	−1.35 (−2.09 to −0.61)	<0.001	72.8 (72.3–73.0)	72.4 (71.9–72.9)	−0.37 (−0.90 to 0.16)	0.068
Healthcare setting								
Primary	135.3 (134.8–135.9)	133.0 (132.4–133.6)	−2.31 (−2.99 to −1.63)	<0.001	75.6 (75.3–76.0)	73.5 (73.1–73.9)	−2.15 (−2.60 to −1.70)	<0.001
Secondary	130.7 (129.5–132.0)	131.7 (130.6–132.9)	1.01 (−0.36 to 2.37)	0.144	66.5 (65.6–67.5)	69.9 (69.0–70.8)	3.38 (2.34 to 4.42)	<0.001
Tertiary	132.5 (131.6–133.5)	132.5 (131.6–133.5)	0.02 (−1.05 to 1.09)	0.877	70.1 (69.4–70.9)	71.6 (70.9–72.3)	1.48 (0.69 to 2.28)	<0.001
BMI (kg/m^2^)								
<18.5	130.3 (124.7–135.9)	130.1 (123.5–136.7)	−0.20 (−7.22 to 6.83)	0.909	65.8 (62.1–69.5)	66.6 (61.9–71.3)	0.78 (−3.46 to 5.02)	0.630
18.5–25	131.7 (130.3–133.0)	131.5 (130.1–132.9)	−0.16 (−1.69 to 1.38)	0.646	70.6 (69.6–71.6)	70.8 (69.8–71.7)	0.22 (−0.89 to 1.32)	0.401
25–30	134.0 (133.1–134.8)	132.4 (131.6–133.2)	−1.56 (−2.54 to −0.57)	<0.001	73.7 (73.1–74.4)	73.0 (72.4–73.6)	−0.74 (−1.42 to −0.05)	0.007
>30	134.7 (134.0–135.4)	133.8 (133.2–134.5)	−0.89 (−1.65 to −0.13)	0.044	74.1 (73.6–74.6)	74.2 (73.5–74.7)	0.07 (−0.48 to 0.62)	0.939
Smoking								
Smoker	133.7 (132.9–134.5)	132.4 (131.6–133.1)	−1.32 (−2.21 to −0.42)	0.004	73.0 (72.4–73.5)	72.2 (71.7–72.8)	−0.75 (−1.37 to −0.14)	0.011
Non-smoker	134.1 (133.5–134.7)	132.9 (132.3–133.5)	−1.19 (−1.85 to −0.53)	<0.001	72.8 (72.4–73.3)	72.6 (72.2–73.1)	−0.23 (−0.70 to 0.24)	0.128

BMI: body mass index; * Wilcoxon signed-rank test.

**Table 3:** Adherence to antihypertensive medication across subgroups at 6-months.

Characteristics	Overall Adherence (n = 2223, 41.9%)	Number to Antihypertensive Medications			*p*-Value*
		One (1764, 42.8%)	Two (395, 39.4%)	Three or More (189, 33.9%)	0.011
Gender					
Men	1032 (42%)	821 (43.1)	180 (39.5)	31 (31.3)	0.211
Women	1191 (41.8%)	943 (42.7)	215 (39.2)	33 (36.7)	0.037
Age (years)					
18–40	364 (67.9%)	326 (68.2)	37 (63.9)	1(-)	0.696
41–50	726 (56.6)	603 (57)	103 (57.2)	20 (44.1)	0.246
51–60	799 (35.7)	609 (36)	163 (34.9)	27 (35.5)	0.912
61–75	222 (24.3)	154 (23.2)	59 (28.4)	9 (20.9)	0.280
>75	112 (33)	72 (32)	33 (36.7)	7 (29.2)	0.668
Health center location					
Rural	1080 (41.6)	850 (42.2)	201 (40.9)	29 (31.9)	0.135
Urban	1143 (42.1)	914 (43.4)	194 (38)	35 (35.7)	0.037
Healthcare setting					
Primary	1280 (40.1)	1024 (41.1)	213 (36.6)	43 (38.1)	0.127
Secondary	390 (49.1)	296 (50.3)	86 (48.9)	8 (25.8)	0.029
Tertiary	553 (41.8)	444 (42.9)	96 (39.3)	13 (28.9)	0.122
Smoking					
Smoker	587 (31.2)	442 (31.4)	125 (31.8)	20 (23.8)	0.067
Non-smoker	1636 (47.8)	1322 (48.8)	270 (44.3)	44 (41.9)	0.326
Body mass index (kg/m^2^)					
18.5–25	297 (47.1)	238 (47.6)	53 (46.5)	6 (35.3)	0.601
25–30	588 (39.6)	462 (40.2)	109 (38.9)	17 (29.8)	0.282
>30	916 (37.8)	733 (38.8)	153 (33.7)	30 (36.1)	0.125
Diabetes					
No	1756 (39.6)	1378 (40.5)	326 (37.8)	52 (30.4)	0.015
Yes	467 (53.6)	386 (54.1)	69 (49.6)	12 (66.7)	0.337

* chi-square test.

**Table 4:** Factors associated with adherence to antihypertensive medications.

Variables	One Medication		Two Medications		Three Medications	
	Crude	Adjusted	Crude	Adjusted	Crude	Adjusted
Age	0.94 (0.93–0.95) **	**0.93** (0.92–0.94) **	0.96 (0.94–0.97) **	**0.95** (0.93–0.96) **	0.96 (0.93–0.99) **	0.96 (0.92–1.01)
Sex						
Men	0.98 (0.86–1.11)	**1.20** (1.04–1.39) **	0.98 (0.76–1.26)	0.95 (0.71–1.27)	1.27 (0.69–2.32)	1.54 (0.75–3.17)
Women	1	1	1	1	1	1
Health center location						
Rural	0.95 (0.84–1.07)	0.87 (0.73–1.04)	1.12 (0.87–1.45)	0.91 (0.63–1.30)	0.82 (0.46–1.54)	0.62 (0.26–1.50)
Urban	1	1	1	1	1	1
Healthcare setting						
Primary	0.92 (0.80–1.07)	0.92 (0.78–1.09)	0.89 (0.65–1.21)	0.89 (0.62–1.27)	1.51 (0.71–3.19)	1.57 (0.66–3.73)
Secondary	1.35 (1.10–1.65) **	**1.45 ** (1.15–1.84) **	1.47 (0.99–2.18)	1.49 (0.95–2.35)	0.85 (0.30–2.40)	0.77 (0.23–2.55)
Tertiary	1	1	1	1	1	1
Smoking status						
Non-smoker	2.07 (1.83–2.37) **	1.12 (0.95–1.33)	1.70 (1.30–2.22) **	1.07 (0.76–1.52)	2.30 (1.22–4.35) **	1.55 (0.62–3.82)
Smoker	1	1	1	1	1	1
BMI (kg/m^2^)	0.99 (0.98–0.99) **	**0.98** (0.97–0.99) **	0.97 (0.95–0.99) **	**0.96** (0.94–0.98) **	1.02 (0.97–1.07)	1.02 (0.97–1.07)
Diabetes						
Yes	1.73 (1.47–2.03) **	**1.27** (1.06–1.53) **	1.62 (1.13–2.32) **	1.15 (0.76–1.73)	4.57 (1.63–12.85) **	**3.61** (1.11–11.72) *
No	1	1	1	1	1	1

Adjusted for age, type of health center, BMI, and smoking. Significant factors were bolded * Significant at *p* <0.05; ** significant at *p* <0.001.

**Table 5:** Age-stratified mean blood pressure in hypertensive patients with TRH and PRH.

	<65 Years		≥65 Years	
	Systolic BP	Diastolic BP	Systolic BP	Diastolic BP
Treatment-resistant hypertension	146.5 ± 13.8 mmHg	91.0 ± 9.7 mmHg	151.0 ± 8.2 mmHg	101.0 ± 4.9 mmHg
Pseudo-resistant hypertension	144.2 ± 11.6 mmHg	92.3 ± 5.8 mmHg	159.5 ± 10.8 mmHg	99.4 ± 8.0 mmHg

## Data Availability

All the data is available is available with the corresponding author and can be obtained with suitable reason.
